# The complete chloroplast genome of *Dendrobium moschatum* (Buch.-Ham.) Sw. 1805 (Orchidaceae)

**DOI:** 10.1080/23802359.2022.2149249

**Published:** 2022-12-09

**Authors:** Yan-Bing Yang, Feng-Xia Yan, Lian-Hui Wang, Fan Tian, Feng-Jiao Zhou

**Affiliations:** a Guizhou Academy of Forestry, Guiyang, China; b Guizhou Institute of Forestry Inventory and Planning, Guiyang, China

**Keywords:** *Dendrobium moschatum*, chloroplast genome, phylogeny

## Abstract

The morphological characteristic of *Dendrobium moschatum* (Buch.-Ham.) Sw. 1805 is very distinctive among *Dendrobium* Sw. 1799, and it has high medicinal and ornamental values. Here, we reported the first complete chloroplast genome of *D. moschatum*. The complete genome of *D. moschatum* was 159,701 bp in length with 130 genes, including 38 tRNA, 8 rRNA, and 84 protein-coding genes. Phylogenetic analysis showed that *D. moschatum* was strongly allied with *D. denneanum* Kerr. 1933.

The genus *Dendrobium* Sw. 1799 (Orchidaceae) is one of the largest genera in angiosperms, over 1500 species that are mainly distributed in Asia and Oceania (Teixeira da Silva et al. 2016). Most members of this genus have high ornamental value and some *Dendrobium* species are widely used in Chinese traditional medicine. *Dendrobium moschatum* (Buch.-Ham.) Sw. 1805 has a very distinctive morphological characteristic among *Dendrobium* species featured with the length of erect stem to 1 m, inflorescences 20 cm, lip slipperlike (Zhu et al. 2009) (Figure 1). However, due to its highly medicinal and ornamental values, the wild resources of *D. moschatum* have been anthropogenically over-exploited. The native population of the species has dramatically declined and *D. moschatum* has been ranked as critically endangered (CR) of the Red List of China Higher Plants based on IUCN Red List Categories and Criteria (Qin et al. 2017). Therefore, to better reserve this orchid and understand its genetic information, we assembled and characterized the complete chloroplast (cp) genome of *D. moschatum*.

The leave samples of *D. moschatum* were collected from Guizhou Dendrobium germplasm bank in Guiyang, Guizhou, China (106.73 E, 26.49 N). Voucher specimens were deposited in the Dendrological Herbarium in Guizhou Academy of Forestry (GZAF, He Li, 1043630529@qq.com, voucher number: 202110045) and were identified as *D. moschatum* by Professor Lian-Hui Wang. The plant sample is cultivated and collection was permitted by the Institute of Forestry Biotechnology, Guizhou Academy of Forestry. Total DNA was extracted from fresh leaves using modified CTAB method (Doyle and Doyle 1987) and sequenced on Illumina nova-seq 6000 platform. Genome sequences were identified and assembled with SPAdes v.3.5.0 (Lapidus et al. 2014). The genome was annotated by CpGAVAS2 (Shi et al. 2019) and GeSeq (Tillich et al. 2017). The circular genome map was drawn using CPGView program (http://www.1kmpg.cn/cpgview/) (Figure 2).

**Figure 1.**
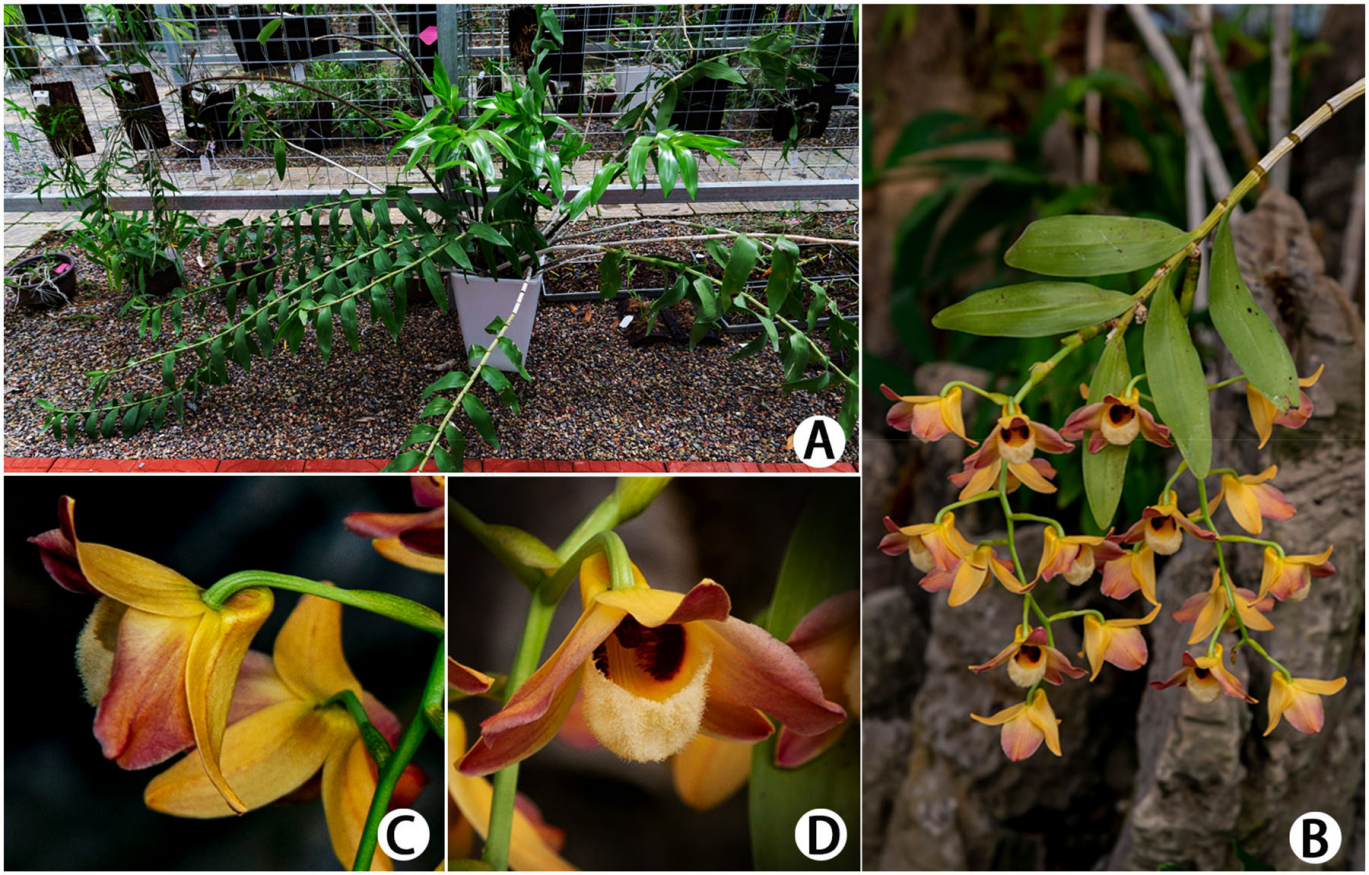
*Dendrobium moschatum*. (A) Plant; (B) inflorescences; (C) lateral view of the opened flower; (D) frontal view of the opened flower.

The cp genome of *D. moschatum* (GenBank accession OM161978) is 15,9701 bp in length, which presented a typical quadripartite structure, containing a large single-copy (LSC: 87,441 bp) region, a small single-copy (SSC: 17,544 bp) region, and two inverted repeat regions (IRA and IRB: 27,358 bp) (Figure 2). Furthermore, 130 genes were annotated in the cp genome of *D. moschatum*, including 84 protein-coding genes, 38 tRNA genes, and eight rRNA genes. The total GC content of the cp genome is 37.26% (Figure 2).

**Figure 2.**
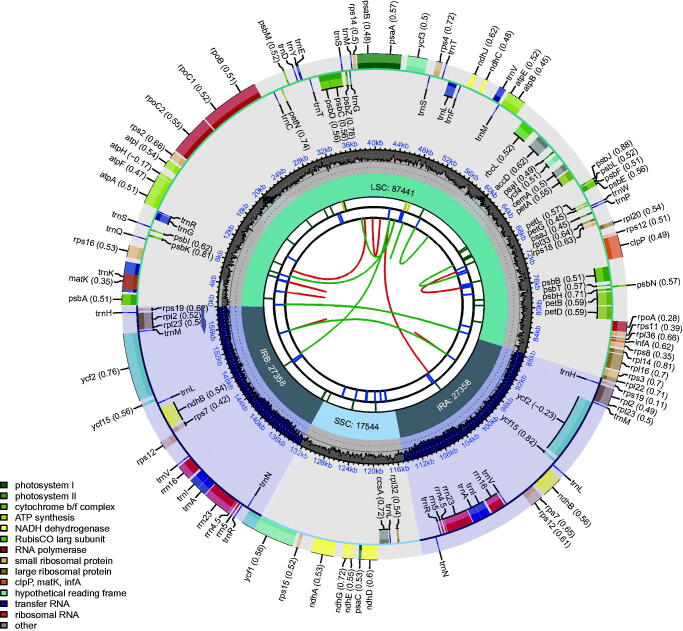
Complete plastome gene map of the *Dendrobium moschatum*. The map contains six tracks. From the center outward, the first track shows the dispersed repeats. The dispersed repeats consist of direct (D) and Palindromic (P) repeats, connected with red and green arcs. The second track shows the long tandem repeats as short blue bars. The third track shows the short tandem repeats or microsatellite sequences as short bars with different colors. The small single-copy (SSC), inverted repeat (IRa and IRb), and large single-copy (LSC) regions are shown on the fourth track. The GC content along the genome is plotted on the fifth track. The base frequency at each site along the genome will be shown between the fourth and fifth tracks. The genes are shown on the sixth track.

To infer phylogenetic position of *D. moschatum*, other 30 *Dendrobium* plastid genomes were selected to carry out analyses with *Paphiopedilum micranthum* T. Tang et F. T. Wang 1951 and *P. armeniacum* S. C. Chen et F. Y. Liu 1982 (Orchidaceae) as outgroups. Sequences were aligned using MAFFT 7.409 (Katoh and Standley 2013), and maximum-likelihood (ML) analysis was performed using RAxML-HPC2 on XSEDE v.8.2.12 (Stamatakis 2014) on the CIPRES Science Gateway (http://www.phylo.org/) (Miller et al. 2010) under the GTRGAMMA substitution model. The result showed that *D. comatum* is phylogenetically related to *D. denneanum* Kerr 1933 (Figure 3). This newly reported cp genome of *D. moschatum* is of great benefit to further investigation on its phylogeny and conservation in *Dendrobium*.

**Figure 3.**
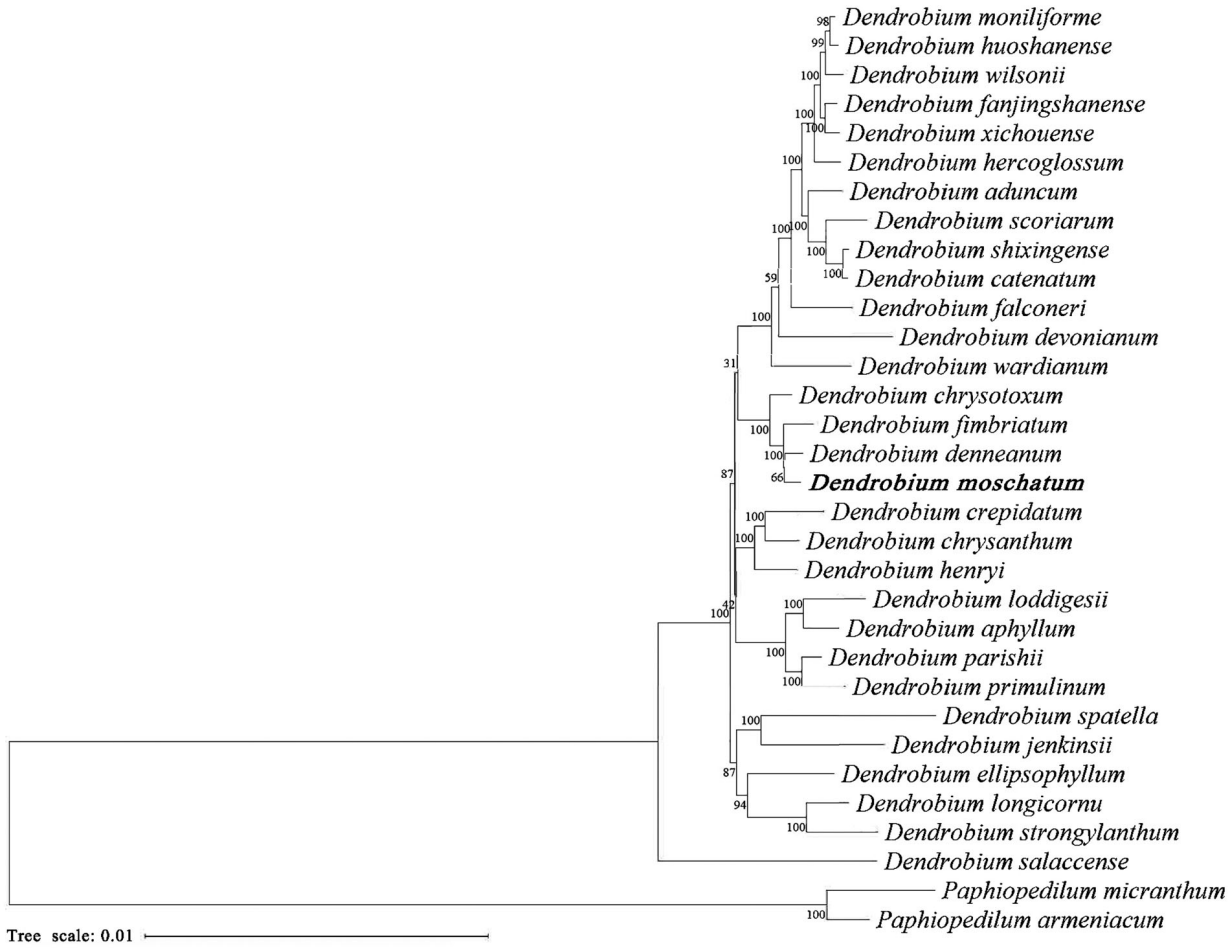
Phylogenetic tree inferred from maximum-likelihood based on 32 complete chloroplast genomes with *Paphiopedilum micranthum* and *P. armeniacum* as outgroups. The position of *Dendrobium moschatum* is marked in bold. The following sequences were used: *Dendrobium aduncum* LC192953 (Zhu et al. 2018), *D. aphyllum* LC192953 (Zhitao et al. 2017), *D. catenatum* KX507360 (Zhong et al. 2016), *D. chrysanthum* LC193514 (Zhitao et al. 2017), *D. chrysotoxum* LC193517 (Zhitao et al. 2017), *D. crepidatum* LC193509 (Zhitao et al. 2017), *D. denneanum* LC192955 (Zhitao et al. 2017), *D. devonianum* LC192956 (Zhitao et al. 2017), *D. ellipsophyllum* LC193519 (Zhitao et al. 2017), *D. falconeri* LC192957 (Zhitao et al. 2017), *D. fanjingshanense* LC193523 (Zhitao et al. 2017), *D. fimbriatum* LC193521 (Zhitao et al. 2017), *D. henryi* LC193513 (Zhitao et al. 2017), *D. hercoglossum* LC192959 (Zhitao et al. 2017), *D. huoshanense* LC269310, *D. jenkinsii* LC193515 (Zhitao et al. 2017), *D. loddigesii* LC317044 (Niu et al. 2017), *D. longicornu* MN227146 (Wu et al. 2019), *D. moniliforme* MN200384 (Kim et al. 2020), *D. parishii* LC193518 (Zhitao et al. 2017), *D. primulinum* LC192810 (Zhitao et al. 2017), *D. salaccense* LC193510 (Zhitao et al. 2017), *D. scoriarum* LC348851 (Zhu et al. 2018), *D. shixingense* LC348860 (Zhu et al. 2018), *D. spatella* LC193511 (Zhitao et al. 2017), *D. strongylanthum* KR673323 (Li et al. 2016), *D. wardianum* LC192961 (Zhitao et al. 2017), *D. wilsonii* LC193508 (Zhitao et al. 2017), *D. xichouense* LC193520 (Zhitao et al. 2017), *Paphiopedilum armeniacum* KT388109 (Kim et al. 2015), and *P. micranthum* NC_045278.

## Data Availability

The genome sequence data that support the findings of this study are openly available in GenBank of NCBI at https://www.ncbi.nlm.nih.gov under the accession no. OM161978. The associated BioProject, SRA, and Bio-Sample numbers are PRJNA838424, SRP375593, and SAMN28422334, respectively.
